# Use of Blood Thinners and Increased Nosebleeds during the Coronavirus Disease 2019 Pandemic

**DOI:** 10.1055/s-0044-1791583

**Published:** 2025-06-10

**Authors:** Lucas Diniz Costa, Ana Paula Brandão Silva, Mariane Stagi Almada, Vanessa Pinheiro Adamo, Guilherme Irie Nakazora, Gustavo Rossoni Carnelli, Antonio Carlos Cedin

**Affiliations:** 1 Hospital BP, Beneficência Portuguesa de São Paulo, São Paulo, SP, Brazil

**Keywords:** epistaxis, nasal bleeding, COVID-19, coronavirus, anticoagulants

## Abstract

**Introduction** In May 2020, the World Health Organization declared Brazil a new epicenter of the coronavirus pandemic.

**Objective** The present study aims to verify if the frequency of nasal bleeding and/or epistaxis in patients of a tertiary hospital was correlated with the pandemic of coronavirus disease 2019 (COVID-19) and with the use of anticoagulants.

**Methods** The analysis performed was retrospective from the database of the otorhinolaryngology service of a Brazilian tertiary hospital, comparing 2 periods: 1 from March 2020 to July 2021 comprising the peak of pandemic setting, and another from August 2021 to May 2022. We checked data on the average number of cases/month and on the use of anticoagulants.

**Results** In the period above mentioned, there were 61 cases of COVID-19-related epistaxis (from a total of 180 cases of nasal bleeding and/or epistaxis), with an average of 12 cases/month, demonstrating an increase in the frequency of cases at the institution, when compared to a study involving 343 cases from the same institution over a period of 42 months (October 2015–March 2019), in which the average was 8.1 cases/month. Among the patients with COVID-19 and nasal bleeding, 55 (90.17%) were using some type of anticoagulant at the time of bleeding: 41% were on subcutaneous heparin; 20% were on subcutaneous enoxaparin; 16.66% were receiving intravenous heparin on continuous infusion bomb (CIB); 6.66% were on extracorporeal membrane oxygenation (ECMO) associated with intravenous heparin on CIB; 4.99% were on oral rivaroxaban; and 1.66% are on oral apixaban.

**Conclusion** Our study's data confirmed the increase in the number of epistaxis cases and the use of anticoagulants in COVID-19 patients.

## Introduction

In May 2020, the World Health Organization (WHO) declared Brazil a new epicenter of the coronavirus pandemic.

## Objective

The present study aims to verify if the frequency of nasal bleeding and/or epistaxis in patients of a tertiary hospital was affected by the pandemic of COVID-19.

## Methods

This is a retrospective review of the epistaxis cases treated at the department of otorhinolaryngology of a Brazilian tertiary hospital, comparing 2 periods: 1 from March 2020 to July 2021, comprising the peak of pandemic setting, and another from August 2021 to May 2022.

All medical consultations were performed on patients aged 18 or older. The patients' data, such as date of admission to hospital, date of birth, age, and date of the epistaxis, were registered in a record book as the epistaxis events occurred. Recurrences of epistaxis and postsurgery nasal bleeding were excluded from this study.

We performed an analysis in our electronic database to check information about the polymerase chain reaction (PCR) COVID status and anticoagulation treatment in use. The collected data were tabulated and accounted for considering the total and average cases/month in each of the two periods above mentioned. Then, we compared the average cases/month of epistaxis between these periods using t-test to assess for statistically significant difference.

Also, we accessed the use of anticoagulants, detailing the type and the dose of each. All data sorting, calculations, and charts were done in Microsoft Excel (Microsoft Corp., Redmond, WA, USA).

The present article is in accordance with chapter IX of the Brazilian Resolution number 674, of May 06 of 2022, which explains about the Ethics Committee in Brazil. According to this resolution, this paper is exempt from verification. (resolution on supplements files).

## Results

In the first period (March 2020–July 2021), there were 180 cases of nasal bleeding and/or epistaxis, resulting in an average of 12 cases/month, demonstrating an statistical significant increase ( *p* < 0.01) in the frequency of cases at the institution, when compared to a study involving 343 cases from the same institution over a period of 42 months (October 2015–March 2019), in which the average was 8.1 cases/month. 1

Of the 180 cases, 61 (33.88%) were patients with COVID-19 (C-reactive protein [CRP]-positive at the time of admission). Of these 61 cases, 55 (90.17%) were using some type of anticoagulant at the time of bleeding: 41% were on subcutaneous heparin (21%: 5,000 UI 3 times/day; 20%: 5,000 UI 2 times/day); 20% were on subcutaneous enoxaparin (3.33%: 40 mg once a day; 1.66%: 60 mg once a day; 1.66%: 80 mg once a day; 1.66%: 100 mg once a day; 8.33%: 60 mg 2 times/day; and 3.33%: 80 mg 2 times/day); 16.66% were receiving intravenous heparin on continuous infusion bomb (CIB); 6.66% were on extracorporeal membrane oxygenation (ECMO) associated with intravenous heparin on CIB; 4.99% were on oral rivaroxaban; and 1.66% are on oral apixaban.

In the second period (August 2021–May 2022), there were 102 cases, and the rate of cases/month decreased to 10.2 cases/month ( *p* < 0.01). Also, the percentage of COVID-19-related epistaxis episodes decreased: only 10.79% of the nasal bleeding episodes were on COVID-19-positive patients.

Table 1

**Table 1:** Number of patients (%) according to anticoagulant therapy in use among epistaxis cases (61)

None	Subcutaneous heparin	Subcutaneous enoxaparin	Heparin IV on CIB	Heparin IV on CIB (ECMO)	Rivaroxaban	Apixaban
6 (9.8%)	25 (41%)	12 (20%)	10 (16.66%)	4 (6.66%)	3 (4.99%)	1 (1.66%)
		**Subcutaneous heparin**				
**Dose**		5,000 UI 3 times/day	5,000 UI 2 times/day			
**Number of patients (%)**		13 (21%)	12 (20%)			
		**Subcutaneous enoxaparin**				
**Dose**	40 mg 1 time/day	60 mg 1 time/day	80 mg 1 time/day	100 mg 1 time/day	60 mg 2 times/day	80 mg 2 times/day
**Number of patients (%)**	2 (3.33%)	1 (1.66%)	1 (1.66%)	1 (1.66%)	5 (8.33%)	2 (3.33%)

**Abbreviations:** CIB, continuous infusion bomb; ECMO, extracorporeal membrane oxygenation; IV, intravenous.

## Discussion

Patients with COVID-19 present with an unregulated systemic inflammatory response and a dysregulated hypercoagulable state associated with arterial and venous thrombotic events. Coagulopathy is characterized by mild thrombocytopenia, mildly prolonged prothrombin time, high levels of D-dimer, and increased levels of fibrinogen, factor VIII, and von Willebrand factor. 2 3

Despite this, in our study, we evidenced an increase in the frequency of epistaxis cases during the new coronavirus pandemic, contrary to the expected logic. Among the hypotheses that would lead to an increase in the number of bleedings, we have greater use of anticoagulants that this population is subjected to, coagulopathy due to consumption, greater use of devices of hospitalized patients (nasoenteral tube, nasal oxygen catheter, non-invasive ventilation). 4 5

Another hypothesis that is worth mentioning is the method to check the PCR COVID status of the patients: nasopharyngeal swabs are the gold-standard and are the method used in our hospital. So, the inappropriate use of the equipment and short time to train the way the health professionals would also contribute to the rise of the nasal bleedings.

Despite the higher number of epistaxis cases, a study from another institution involving 4,389 COVID-19 cases shows that the rate of severe bleeding is low (2%). 6 Thus, given the high mortality of patients hospitalized with COVID-19 with severe coagulopathy, there is an uncertainty of what the ideal anticoagulation regimen to reduce morbidity related to nasal bleeding and, concurrently, reduce mortality would be.

We also noticed a concomitant decrease in the number of epistaxis cases after the COVID-19 pandemic regression (months from August 2021–May 2022). The increase on the average of population vaccinated led to a hard drop in the numbers of hospitalized patients and, therefore, it would also be correlated to decrease of the numbers of COVID-19-related epistaxis. 7

## Conclusion

It is evident that the COVID-19 pandemic contributed to the increase in the number of hospital admissions, and the present study demonstrates the impact on the increase in the number of epistaxis cases in hospitalized patients. Prospective studies to develop a safe anticoagulation protocol are needed to reduce the number of bleedings in patients with COVID-19.
